# Criticisms on the Treatment of the Venereal Disease

**Published:** 1800-05

**Authors:** T. Vage


### *Criticisms on the Treatment of the Venereal Disease. By* T. Vage, M. D.

To the Editors of the Medical and Physical Journal.

Gentlemen,

THE dreadful effects of the venereal virus on the human frame is too well known to need description; and from the numerous examples of them, which every where occur, some may presume that our knowledge in this respect is very defective. Few diseases, however, are more within the improvements of medical skill, if timely taken; and all its difficult, or desperate cases, arise either from injudicious treatment in the beginning, or from a total neglect. Proficiencies in medicine, it is to be regretted, are tedious in reaching the bulk of practitioners; habits in the profession, like all other habits, are tenacious of perseverance: and an instance or two of fancied success, which frequently arise from nature or constitution, are enough to sanction a general failure from any suspicion or innovation. Indeed, a great number of young men are bred up to medicine, without any regular precepts, and grow old, without the benefits of experience. What renders this observation vation of more importance is, that a great part of the faculty, from being immediately connected with the lower classes of the people, are sure to have the greatest number of cases of this, as well as of other diseases, under their care. Hence a ready means of diffusing medical improvements, would be almost as useful as improvements themselves. The Medical and Physical Journal, from its general reception, is likely to answer this purpose; and if the following criticisms are conducive to the plan of that publication, you will no doubt, when convenient, give them a place in it. They are chiefly addressed to gentlemen of the above description; not intended as a methodical instruction, but merely to remark some leading errors, and to shew the necessity of consulting, more minutely, authors who have written the improvements of practice in detail. Practitioners of information will excuse here, what they know already, and receive what appears novel as the result of practical attention. As I purpose, in a future paper, to send some remarks on confirmed lues, and on the means of restoring constitutions shattered by mercury, or a debauched life, I shall, in this, confine myself to the local and inflammatory stages of the disorder.

The improvements in the cure of syphilitic complaints, have chiefly turned on different preparations and modes of using the grand specific, mercury. Many other things have been, at different times, invented, which credulity in some, and the crafts of repute in others, procured their day of vogue; but they have all relapsed into disuse. Mercury is the only one that has stood the test of time; yet it is certain, that though it is effectual in subduing the disease, it often breaks down a constitution, and subjects it to a variety of nervous and chronic infirmities. A medicine therefore with its efficacy, and without its alloy, would be a valuable discovery. The nitric acid, the topic of the animated controversy of the present time, seems not to gain ground; and my own experience inclines me to think, it will reflect no great honour on the new doctrines from which it is derived.

Some contagious diseases, even the plague itself, have natural limits to their progress of corruption, which the strength of constitution is frequently able to surmount. Others, among which syphilitic infection is one, have a sure though gradual progress to destruction; and which medical skill alone is capable of preventing. Old practitioners appear to have had but a rude conception of this disease, and to have instituted such a general mode of treatment, that, when it came to be distinguished into its proper stages, was not, scientifically, applicable to any one of them. Thus, for instance, in a recent gonorrhœa, the most moſt violent remedies were choſen, ſuch as ſtrong alternate doſes of mercury and cathartics, and even ſalivation, &c. which indeed might have in general ſucceeded, but left many a good conſtitution irrecoverably impaired: Nor is this dangerous practice yet entirely aboliſhed, although it is well underſtood by many, that very ſuperficial means are commonly ſufficient for the buſineſs; for what indiſpoſition or inconvenience can ariſe from a mild mercurial injection, a few proper purges, a little nitre, with ſome emollient drinks, ſuch as linſeed tea and the like, or, occaſionally, an article or two more, as gentle as the former?

But in diſcovering errors of importance, there is often a propenſity to run into oppoſite extremes. On finding ſuch incipient infection to be chiefly of a local nature, it was immediately conceived, that the introduction of mercury into the habit was altogether needleſs. And if I miſtake not, ſome have gone ſo far as to think, that a gonorrhœa is incapable to produce a confirmed lues. But let it be conſidered, that this contagious poiſon generates itſelf very rapidly; that it begins its aſſimilation the moment it inflames a part; and that an abſorption of the infection, from its local affections, is the common origin of its progreſs. What is a gonorrhœa, or any of the firſt inflammations, but the infectious matter in operation?—And who, that knows any thing of the ſtructure of our ſolids, will aſſert that ſuch caſes can be deſtitute of abſorption of ſome of the infectious matter into the humors?—In the ſlighteſt caſes, therefore, this ought to be ſuſpected; and as the adjacent glands may be conſidered as the next ſteps of the diſeaſe, and the lymphatics the conveyances of the infection, ſome ungt. hydrarg. ought to be rubbed into the lower extremities, at proper intervals, and round the glans penis, in order to counteract its effects. In this intention, modern practice ſtill admits, for a time, the internal uſe of ſome mercurial preparation in alterative doſes, ſuch as calomel, mercur. calcinat. &c. and as it threatens no hurt to the conſtitution, and is provident of future ſafety, a criticiſm on its propriety may be well ſpared.

Salivation, in the advanced ſtages of the diſorder, ſtill retains its repute with ſome men of eminence; but the inconſiſtency of it, with the true deſign of cure, will evidently appear, when it is conſidered that the ſeats of the diſeaſe here are deeply fixed on the glands, perioſteum, bones, membranes, &c. and that mercury, this way, never gets ſufficiently out of the great channels of circulation, to pervade ſuch diſtant parts. For whatever paſſes into the blood, which is not of a nutritious nature, acts as an extraneous ſubſtance, diſcompoſing the animal economy, and irritating it until it is expelled, by one or or other of the emunctories, which are placed in different parts of the body for that purpose. Mercury naturally affects the salival glands; and it was this, perhaps, that first suggested the idea of employing it this way. To have its specific and remote effects, therefore, it must act insensibly, or, in other words, it must be given in alterative doses.

A very important defect in the treatment of gonorrhoea arises from strictures and caruncles, which frequently remain in the urethra after cure, as they lay the foundation of future fistulæ, or the necessity of the painful introduction of escharotic bougies. If any errors of ignorance are unpardonable, this is one; because it is easily prevented by proper bougies during the cure, when the inflammatory symptoms subside, and the ulcerations begin to heal; for then fungous flesh soon protuberates, becomes callous, and choaks up the passage. Testicular inflammations also are commonly the consequence of injudicious practice, and especially by astringent injections and strong cathartics; for it is well known, that a diminution or stoppage of the discharge is suddenly productive of this effect; and yet I find that a solution of zinc. vitr. as an injection in recent cases, is at this very time used by some practitioners of note. Facts, however, warrant me to observe, that it is only some fortunate circumstances, or corrective counteraction, that can prevent its mischief. This remedy can be only serviceable in the relaxations which remain after violent methods of cure, or are induced by debilitated habits of body. Another common oversight here, is the want of a frequent change of linen cloths, to absorb and remove the discharge; this cannot fail to retard the cure considerably; for if the contact of such virulent matter would infect a sound person, it must, in some degree, keep up a renewal of the infection in a patient.

Mild mercurial injections (among which the simplest one of good calomel and water is eligible) are leading remedies of improved practice; yet, when the inflammation runs high, something to accelerate their effects appeared wanting. The speedy ease which acetated lead affords in external inflammations, and its safety in so sensible a part as the eye in opthalmia, would seem to favour this design, were it not for the strong astringency commonly ascribed to it. I resolved, however, on a cautious trial, and mixed a portion of cerus. acetat. with the simple injection above mentioned; the effect was an abatement of pain: the discharge was indeed diminished, but nothing more than might be reasonably attributed to the diminution of the irritation. From this, I began to think, that the efficacy of this metal did not arise from any astringent property; and what corroborated this idea was, that emollient and relaxing applications cations, alleviate the ſame painful ſymptoms for which lead is commended; and I had the curioſity to inquire for a principle of action leſs objectionable.

### *Theory of the Properties of Leaa.*

The moſt certain criterions of the qualities of any thing, would appear to be the exceſs or continuance of its operation. In painters, who are conſtantly in the uſe of white lead, in workmen employed in its preparation, and in people who are accuſtomed to drink cyder which has been made in leaden utenſils, we have evident proofs of the deleterious effects of this metal; and they are all of a nervous, debilitating nature: palſies, tumours, ſpaſms, inteſtinal conſtipations, and the like, and even intermiſſions of the muſcular actions of the circulatory functions. But all theſe effects are referable to one and the ſame cauſe, a diminution of the nervous influence, producing leſs ſenſibility in the parts. Hence is clearly deducible the topical utility of this remedy, in the abatement of pain in inflammatory ſymptoms, its repreſſion of hæmorrhages, and, indeed, all that has been uſually aſcribed to an aſtringent power; for inflammation ariſes from the ſenſibility of the muſcular fibres, irritated by ſome pungent cauſe, which exciting inordinate and convulſive motions in theſe fibres, extravaſate the vaſcular fluids into the adjacent interſtices: ſo that lead, by ſuſpending the ſenſibility of an inflamed part, muſt conſequently eaſe pain, and ſtop the progreſs of the inflammation. The periſtaltic motion of the inteſtines is impeded the ſame way, and obſtinate coſtiveneſs occaſioned. If this theory is juſt, it would ſeem to open a field for further uſeful applications of this remedy.

Sometimes the firſt appearance of the diſeaſe is in ulcers of the glans, prepuce, frenum, &c. and theſe are often ſo painful and obſtinate, as to induce ſome to ſuppoſe the infection to be deep rooted. This idea has often ſuggeſted the neceſſity of a tedious and injurious exhibition of mercury. But the obſtinacy of theſe caſes, as it is acknowledged even at this time by many practitioners of eminence, permits us to conclude that practice, here, ſtill admits of conſiderable improvement, at leaſt in an eſtabliſhed way. Nor does it require deep reflection to know, that the ſtubbornneſs of theſe ulcers ariſes immediately from the morbid fleſh contiguous to their ſurface; and that, of courſe, a direct and deciſive deſtruction of ſuch infected parts, (as remarked in a former paper on inveterate ulcers) cannot fail to reduce them to a healing ſtate. The ſooner this is done, the more it will expedite the cure; for while the generation of infectious matter continues, not only ſome abſorption of it, but the local cauſe itſelf of the obſtinacy of theſe ſores will continue to increafe alfo. A folution of hydrarg. in the nitrous acid, of a proper ftrength, very effectually anfwers that end, with a convenient cataplafm next to the furface of the ulcers, to caft off the floughs. In fuperficial cafes, dreffings of ungt. hydrarg. nitrat have been found fufficient. When the cure of thefe ulcers is tedioufly attempted by the internal ufe of mercury, the inguinal glands feldom efcape infection; but this may, in general, be prevented by a friction of ungt. hydrarg. into the lower extremities, &c. as mentioned in the gonorrhœa.

Ulcers of the mouth, throat, and palate, &c. although commonly as local as the former, are vaftly more dangerous, both from the importance of thefe parts, and from the infection paffing into the alimentary canal, and corrupting the very fources of nutrition and mefenteric glands. This happens oftener than is obferved, and is capable of producing a confirmed lues in a very fhort time. Two examples of it I met with fome years ago, from a country infimary, by miftaking fuch ulcers for a common ulcerated throat. Here, an internal ufe of mercury is indifpenfibly neceffary, and a folution of hydrarg. muriat. is a modern remedy of great efficacy, both as a gargle, and given alteratively in a mucilage of g. arab. This medicine is very quick in checking venereal infection, but it has been obferved not to be radically effective. When it has anfwered its purpofe, therefore, fome other preparation of mercury ought to be adopted, to perfect a cure. A great difadvantage of a long ufe of this remedy, and which has perhaps efcaped notice, is, its fubjecting the body to obftinate rheumatic complaints. Here, I beg leave to recommend a folution of hydrarg. in the nitrous acid, to be ufed properly diluted, like the former, in both indications. Much experience enables me to conclude on its fuperior efficacy, both in the recent and advanced ftages of the difeafe. An eligible mode of giving it, is by forming the cryftals, which a faturated folution by heat runs into upon cooling, into pills with fome extract. cort.

Venereal inflammations and abfceffes have many other peculiarities, which modern practice feems not fufficiently to difcriminate. Thus, for inftance, a refolution of buboes is held by fome as good practice, and even a perfect cure, if no other fymptoms remain: but a latent infection is, thereby, fent into the habit, which is pretty certain, one time or other, to break out into a deftructive inveteracy. Others, who, with more propriety, prefer the fuppurative way, err afterwards frequently, by fuffering the abfceffes to break of themfelves. For, befides the abforption from the infectious matter, the retention retention of it beyond its proper time of difcharge, always renders the ulcers deep, foul, and corrofive. Nature, fometimes points too high in thefe cafes, and furgeons are hereby mis-led to operate fo too: thus, fome of the difcharge is, from time to time, fuffered to lodge in the lower part of the cavity, and produces other abfceffes lower down. Buboes are frequently very difficult to heal, like fhankers; and this is thought by fome, to proceed from a general taint: indeed, it is, I believe, imagined by others, that venereal ulcers cannot be healed, while the habit is infected. But dangerous conclufions are likely to be drawn from fuch notions; for nothing is more certain than that they can be brought to a healing ftate, when the diforder is far advanced; and then their healing will be fuppofed a depuratory fign, and confequently will indicate a difcontinuance of medicine where it is neceffary, or a tedious courfe of it where it is not. In all cafes, the fooner fuch ulcers are healed the better; for it is abfurd to think, that a fore, which is generating infection, can purify the conftitution; and when it is healed, one fource of that infection becomes cut off.

Inflammations of the penis are very apt to affume a gangrenous difpofition, perhaps from the peculiar irritability of the part, efpecially from fyphilitic acrimony. Cafes of this tendency often occur in phymofis and paraphymofis, from the virulence of the matter of ulcers in the glans, or internal furface of the prepuce, &c. particularly, when it happens to be naturally too tight. Improved practice, if timely employed, would, perhaps, prevent fuch alarming fymptoms; yet, if called in, it may, from profeffional delicacy, coincide with excufe, and ftill effect much: if, however, not called in, the next error will be, a timid delay in dividing the prepuce, and fcarifying the cryftalline ftrictures: with proper fomentations and cataplafms, I have often feen this fucceed, even where livid fpots and gangrenous veficles had made their appearance.

